# Cow Behavior Recognition Based on Wearable Nose Rings

**DOI:** 10.3390/ani14081187

**Published:** 2024-04-15

**Authors:** Wenhan Feng, Daoerji Fan, Huijuan Wu, Wenqiang Yuan

**Affiliations:** School of Electronic Information Engineering, Inner Mongolia University, Hohhot 010021, China; 32156061@mail.imu.edu.cn (W.F.); wuhuijuan@imu.edu.cn (H.W.); 32256087@mail.imu.edu.cn (W.Y.)

**Keywords:** feeding and rumination, behavior recognition, cow behavior, deep learning

## Abstract

**Simple Summary:**

Currently, smart devices for cows on the market are mainly leg rings and collars, but the behavioral data provided by these devices do not well reflect the real behavior of cows. Therefore, a cow-behavior-detection device based on a wearable device is proposed. It is a set of equipment that collects data on the daily behavior (eating, rumination, other behavior) of cows, which can help people monitor the health status of cows more accurately. This paper proposes for the first time an electronic device worn on the nose of a cow to record real-time behavioral data of the cow. Through these data, the daily behavior of cows can be analyzed, such as the time spent eating and ruminating that day, the number of rumination chews, etc., which can better help farm managers understand the health status of cows, reduce the occurrence of diseases, and thereby improve the overall health of the cows’ welfare. The wearing position of the device has no adverse effects on the normal life of the cows and is suitable for long-term wearing. This equipment helps improve the welfare of dairy cows and has far-reaching value for the dairy farming industry.

**Abstract:**

This study introduces a novel device designed to monitor dairy cow behavior, with a particular focus on feeding, rumination, and other behaviors. This study investigates the association between the cow behaviors and acceleration data collected using a three-axis, nose-mounted accelerometer, as well as the feasibility of improving the behavioral classification accuracy through machine learning. A total of 11 cows were used. We utilized three-axis acceleration sensors that were fixed to the cow’s nose, and these devices provided detailed and unique data corresponding to their activity; in particular, a recorder was installed on each nasal device to obtain acceleration data, which were then used to calculate activity levels and changes. In addition, we visually observed the behavior of the cattle. The characteristic acceleration values during feeding, rumination, and other behavior were recorded; there were significant differences in the activity levels and changes between different behaviors. The results indicated that the nose ring device had the potential to accurately differentiate between eating and rumination behaviors, thus providing an effective method for the early detection of health problems and cattle management. The eating, rumination, and other behaviors of cows were classified with high accuracy using the machine learning technique, which can be used to calculate the activity levels and changes in cattle based on the data obtained from the nose-mounted, three-axis accelerometer.

## 1. Introduction

Abnormal behavior in cows may indicate problems related to their physiological health; as such, the use of automated sensors that record cow behavioral data has become increasingly important. This has prompted the development of a novel device, which is designed to be attached to the cow’s nose for accurate behavior data collection. Certain physiological behaviors and reduced sleep may be caused by inflammation in dairy cows [1].

There exists widespread threats to the welfare of grazing ruminants, which may come from factors such as gastrointestinal upsets caused by feed or a sudden drop in temperature, which can cause illness, and such health issues can manifest in observable changes in behavior, such as reduced feed intake, altered rumination patterns, or increased lethargy, which serve as early indicators of potential welfare concerns [2]. These threats are often reflected in the behavior of ruminants. For example, calves with respiratory disease may present abnormal lying and standing behaviors [3], and calves with diarrhea will lie down and be inactive for longer periods of time [4]. There is also a certain correlation between the lying behavior of cows and their postpartum health [5]. For most, other than the managers of large-scale breeding programs, it is impossible to pay attention to the behavior of cows for a long period of time. In response to this problem, certain dairy cow physiological behaviors are studied and analyzed in this study.

Systematic studies conducted on cow behavior thus far can be roughly divided into two types: those involving video surveillance and deep learning and those utilizing wearable sensors. Most studies on cow behavior have used cameras to collect cow image information and identify cow behavior. Fewer studies have used sensors to collect cow behavior information and perform deep learning processing, and those that do exist are still in their infancy.

Recent advancements in the field of agricultural technology have seen the application of machine learning techniques to analyze and understand the behavior of cows in a detailed manner. For instance, methods such as YOLOX and Siam-AM have been utilized to extract the skeletal features of cows for identification purposes [6,7,8]. Furthermore, the installation of tags on cows enables the measurement of acceleration data through the use of convolutional neural networks (CNNs) [9,10]. A cow’s rumination, eating, and activity behaviors can be analyzed through measured acceleration data [11,12]. The deployment of computer vision—specifically, non-contact video monitoring—facilitates the detection of respiratory behavior in cows and also showcases the efficacy of non-intrusive monitoring techniques [13]. In addition, the detailed monitoring of the eating behavior of group-raised cows, including variables such as the number of eating times, average eating duration, average eating interval, and total eating time, has been documented, thereby highlighting the importance of a precise behavioral analysis in agricultural settings [14]. The application of computer vision for the purpose of extracting discriminant features from the body part coordinates of cows further supports the identification of estrus periods, thus demonstrating the utility of machine learning in reproductive management [15].

Ultra-high frequency radiofrequency (RF) waves have been used to collect information from neck-mounted sensor tags equipped with accelerometers in order to assess the behavior of cows [16]. In addition, non-invasive sniffing methods have been used to accurately measure methane emissions [17]. Acoustic sensor technology has been used to non-invasively monitor cattle vocalizations [18], as well as to automatically identify and classify the feeding behavior of cow sounds [19]. The detection of hoof lesions in cows has been achieved through sound analysis [20]. The classification of the chewing and rumination behavior of cows has been achieved by using sound signals and machine learning [21]. The detection of cow eating behavior and activities has been delivered through earhook sensors [22]. Furthermore, the real-time body temperature of cows has also been detected [23]. There have also been studies on the detection of lameness in cows using certain pedometers and three-axis acceleration sensors [24,25] or to detect differences in eating behavior through lameness [26,27]. Pressure sensors can effectively sense the mandibular movement of cows, in order to detect their basic behavior [28,29,30], and collar-mounted three-axis acceleration sensors have also been used to classify cow behavior [31,32,33].

In this work, we develop a behavioral data collection device that is designed to be worn on the nose of cows. Although the data used in this study were still obtained using the aforementioned three-axis acceleration sensor, the difference between the device in this study and those in previous research is that, for the first time, behavioral data can now be collected from the nose of a cow. Moreover, the proposed device is more accurate than the traditional method for detecting the rumination, feeding, and other behaviors of cows, as it records these behaviors of cows more clearly than when using behavioral data alone.

## 2. Materials and Methods

### 2.1. Animal Housing

**Figure 1:**
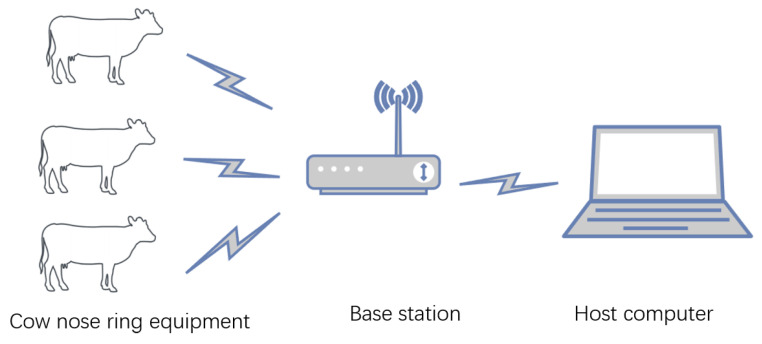
A block diagram of the system’s structure.

This experiment was carried out from 3 June 2023 to 7 June 2023 and 11 June 2023 to 16 June 2023. The data were collected from the cows owned by farmers in Xuniban Village, Hohhot City, Inner Mongolia for 11 days. Data were collected from one cow at a time, for 5–6 h a day; furthermore, some of the data were collected on numerous occasions from certain cows. A total of 7 cows participated in the experiment. The overall block diagram of the system is shown in Figure 1. The reason for selecting seven cows for data collection in this experiment was primarily to assess the correlation between data collected by the cow nose rings and specific cow behaviors such as feeding and rumination. Since these behaviors are generally similar among cows and do not exhibit significant differences, using a larger sample would have likely yielded redundant results and increased experimental costs without enhancing the scientific value.

**Figure 2:**
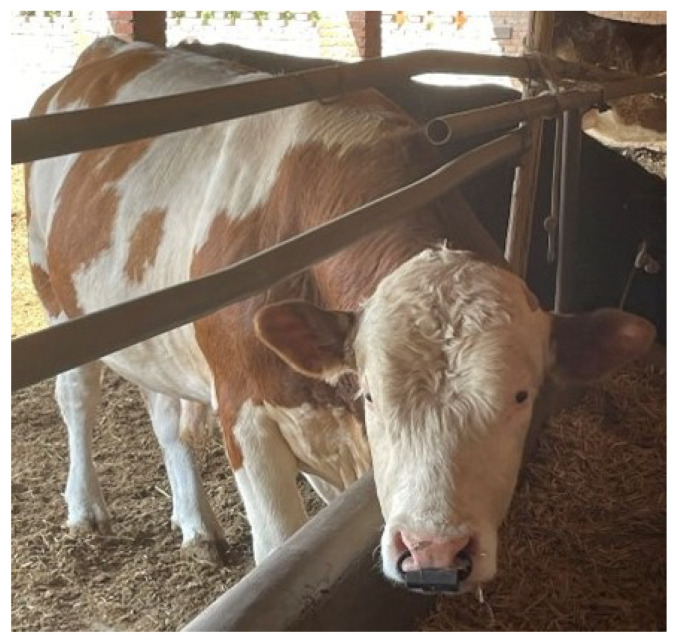
A cow wearing the proposed equipment.

The cows had feeding areas and free movement areas in their homes. Cows are easily frightened when they see strangers, which can lead to irregular activities. Therefore, the farmers chose to set up cameras outside the site for filming. The cows were outfitted with nose rings, the physical representation of which is depicted in Figure 2.

#### Hardware Design

**Figure 3:**
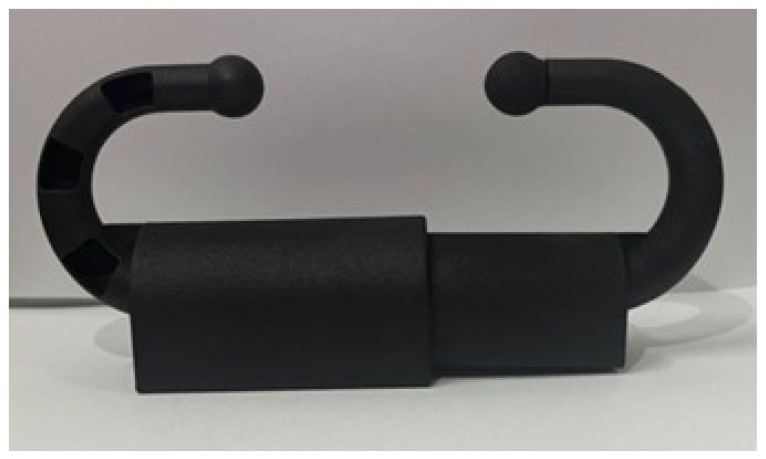
The cow nose ring shell.

The nasal ring device design integrated various components to facilitate efficient real-time data collection and transmission. The microprocessor served as the central processing unit, which managed data collection, preliminary data processing, and coordination with the LoRa module for the purpose of data transmission. The accelerometer captured motion data, which were crucial for analyzing cow behaviors such as feeding, rumination, and other behaviors. The LoRa wireless transmission module was a critical component, as it enabled long-range, low-power communication between the data collection nodes and also aided in the creation of a central data repository or control center. The power module ensured a consistent energy supply for the operation of the device. The described setup employed a nose ring that was equipped with a wireless sensor to capture tri-axial acceleration information from the cow’s nasal region. These data were wirelessly transmitted via a module to a LoRa base station and were then serially sent to a supervisory control system. The device shell is shown in Figure 3.

1. Microprocessor: The STM32L051K8 microprocessor was selected for its low degree of power use, which is ideal for wearables and remote sensors. The STM32L051K8 microprocessor is manufactured by STMicroelectronics, which is headquartered in Geneva, Switzerland. It features a 32 MHz ARM Cortex-M0+ CPU, 64 KB flash, 8 KB SRAM, and operates with a power usage between 1.8 and 3.6 V. In addition, it also supports multiple power-saving modes; as such, it can reduce power consumption according to the user’s preference.

2. Accelerometer: The ADXL362 accelerometer, known for its energy-efficient, three-axis MEMS design, was selected due to its minimal power requirements. The ADXL362 is manufactured by Analog Devices, Inc., headquartered in Wilmington, MA, USA. Its FIFO feature minimizes the microprocessor’s data-saving work, thus resulting in extending low-power sleep cycles and saving energy. It has a 512-sample FIFO capacity that can store large quantities of X, Y, and Z data, thus enabling continuous data collection while the microprocessor is asleep.

3. Communication chip: The SX1278 chip, from Semtech’s SX127x series, was selected for its efficient, long-range LoRa communication capabilities as it outperforms traditional RF methods with a range of 137–525 MHz and is capable of exceeding 10 km. The SX1278 chip is manufactured by Semtech Corporation, headquartered in Camarillo, CA, USA. It also includes error coding for reliability, a 256-byte data packet engine with CRC, and automatic RF detection with RSSI. In a LoRa network, differentiating multiple devices is straightforward via assigning unique device numbers; this allows for simultaneous communications to be queued and processed in order by the base station. We set the transmit power of the SX1278 chip to −4 dBm, the spreading factor to 256, and the channel frequency to 2.4 GHz. Movement changed the communication distance between the two nodes. The communication distance was set to 20, 40, 80, 100, 120, and 150 m. In addition, 300 data packets were sent over a certain distance for testing. Please see Appendix A Table A1 for test data.

4. Power module: The power module for the cow nose ring used a 3.6 V, 2450 mA Saft14500 lithium battery, which was chosen due to its high energy density. The battery is manufactured by Saft, which is headquartered in Levallois-Perret, France. This allowed it to hold more energy than other batteries of a similar size, thus providing durable power without added bulk or weight. This battery ensured that the device could operate continuously for 5–6 months.

**Figure 4:**
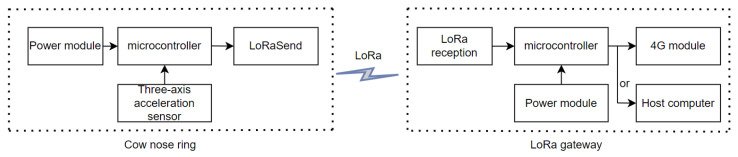
A diagram of the circuit block hardware.

The proposed system was aimed at minimizing power consumption while ensuring continuous data collection and transmission, which was achieved through carefully selecting the microprocessor, accelerometer, communication chip, and power module. This aligns with the objective of efficient and sustainable livestock monitoring in large-scale dairy farming operations. The hardware circuit block diagram is shown in Figure 4.

### 2.2. Data Set Establishment and Classification Model Design

#### 2.2.1. Definition of the Behaviors

In this study, cattle behavior was divided into three categories, namely, feeding, rumination, and other behaviors. Other behaviors were understood as including all behaviors except feeding and rumination. Their definitions are detailed in Table 1.

#### 2.2.2. Data Acquisition and Pre-Processing

**Figure A1:**
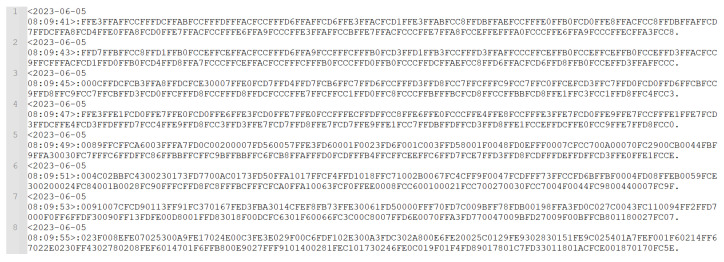
Raw data.

The activity level of 7 healthy cows in the same free-moving breeding area of the study site farm was tested for 11 days in order to verify the detection performance of the nose ring device. A single cow wore the device every day from 8 am to noon. The data for each cow included approximately 113,865 groups (one X, Y, Z axis acceleration represents one group), with a total of 1,252,522 groups. The behavior of the cows was recorded via cameras, which were time-synchronized with the wireless sensors. The camera settings allowed for real-time synchronization, so the recording time matched the actual time. Data from the cow nose rings were uploaded to a base station, which then transmitted them to a supervisory control system for reception. Upon reception, the data were timestamped to ensure that the video recording time aligned with the data upload time. Since the video was recorded continuously, there were no gaps in the video data. See Appendix A Figure A1 for raw data.

If there was any loss of cow behavior data, the extent of the missing data was determined manually. If a significant quantity of behavior data were missing, data annotation was halted until new behavior data that synchronized with the video time became available. The entire process involved meticulous observation by researchers who watched the video alongside the cow behavior data for accurate annotation.

If the data loss was minor, such as the loss of a single set of XYZ three-axis acceleration data corresponding to 1 ms of time, it was deemed insignificant to the experiment, and data annotation continued. Once data annotation was complete, a portion of the annotated data was selected to plot the three-axis acceleration data charts. These charts were reviewed alongside the video to ensure that the data annotation was accurate. Both feeding and rumination behaviors in cows were similar; hence, the patterns in the three-axis acceleration charts remained consistent, which assisted in verifying the accuracy of the data annotation through a visual observation of the charts and video. After the data were annotated, the acceleration time series was cut into same-length segments, and then experiments were performed on the feature value extraction and behavior classification recognition on each data segment.

In this study, we meticulously segmented the original acceleration data into time-length units to enhance the precision and efficiency of the cow behavioral posture recognition, where data completeness, discriminability, and computational efficiency were prioritized in order to find the optimal segment length. After evaluating different duration periods (i.e., 3.2, 6.4, and 12.8 s) for their impact on classification accuracy, 6.4 s emerged as the optimal length for a single data segment.

A 6.4 s segment was found to effectively capture cow behavioral changes as it included sufficient action sequences for accurate behavior differentiation. Segments of 3.2 s may fail to encompass all of the features of certain behaviors, especially complex or longer actions like rumination or partial resting, and this could lead to incomplete data and lower recognition accuracy. Conversely, 12.8-s segments could blend multiple behaviors into one segment, thus complicating classification and diminishing recognition accuracy.

**Figure A2:**
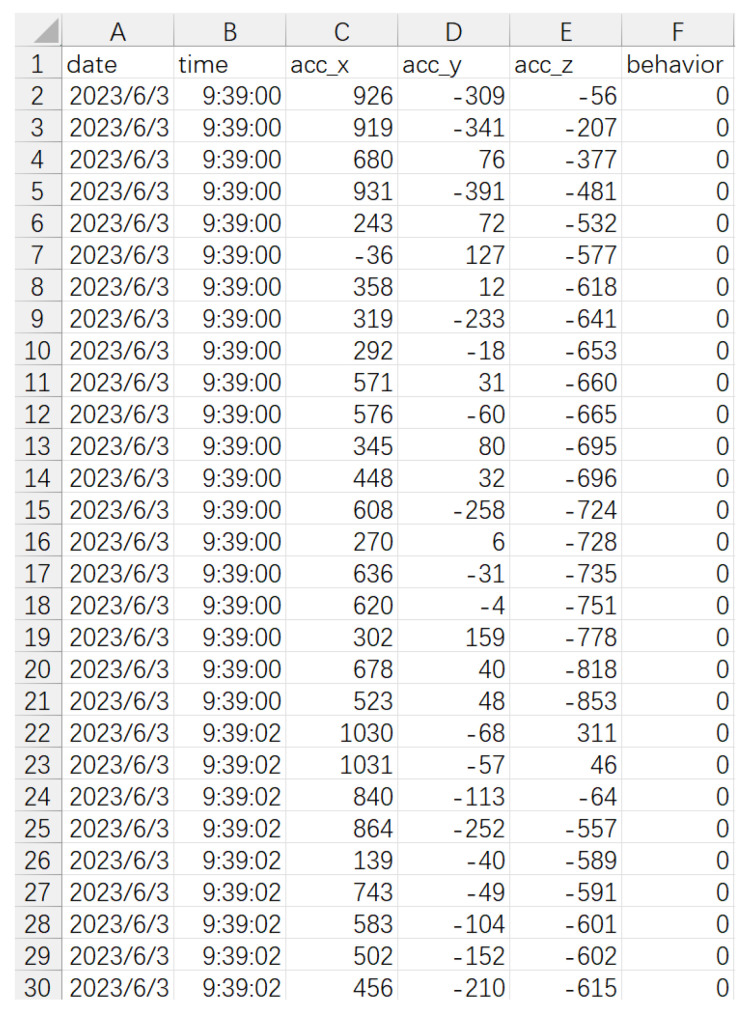
Processed data.

From a computational efficiency and real-time performance standpoint, 6.4 s segments balanced data integrity and discernibility with processing efficiency. While longer segments (e.g., 12.8 s) might reduce data volume, they could also introduce latency in real-time applications, thus affecting system responsiveness and monitoring capabilities. Meanwhile, 6.4 s segments provided a compromise by ensuring accurate behavior recognition alongside swift processing and real-time feedback. Please see Appendix A Figure A2 for the processed data.

The segmented motion data formed the feature vector X, with the corresponding cow behavior posture serving as the target label Y.

Among them, the behavior feature represented the behavior of the cow under that acceleration. The corresponding behaviors were designated in numbers, as shown in Table 2.

**Figure 5:**
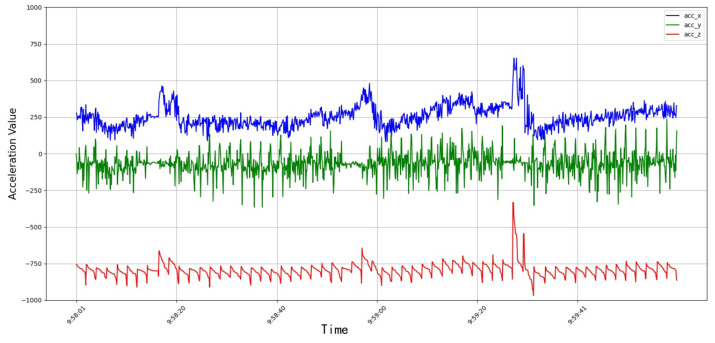
Rumination acceleration curve.

The data set was published at https://www.kaggle.com/datasets/fandaoerji/cow-nose-ring-data-set (CNRD), accessed on 1 November 2023. The acceleration curve of the rumination behavior is shown in Figure 5.

**Figure 6:**
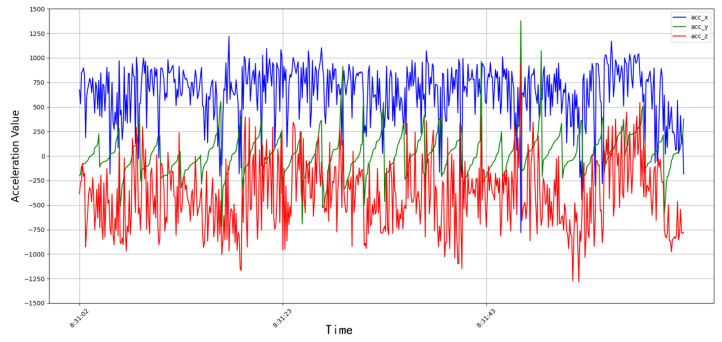
Feeding acceleration curve.

We also observed significant differences between the different behaviors in the distribution range and change amplitude of the acceleration data. The acceleration data of the feeding behavior were distributed in a wide range and varied greatly, which may be related to the frequent head movements and body position adjustments of the cows during feeding. In contrast, the acceleration distribution of the rumination behavior was relatively concentrated, thus indicating that the movements of the cows during rumination were relatively stable and were mainly limited to the rhythmic up and down movement of the head. For the other behaviors, the amplitude of the acceleration changes was not only greater than that of the feeding and rumination behavior, but also changed irregularly, thereby reflecting that the acceleration changes in the cows during other behaviors were more dramatic and changeable. The acceleration curve of the feeding behavior is shown in Figure 6. The length of time a cow takes to eat can be determined by analyzing the Y-axis acceleration, and the feed intake of a cow in a day can be determined based on information such as the length of time and feed weight.

After the cows wore the device, the researchers collected the video of the cow from that day and then annotated the real-time behavior of the cow with the collected cow behavior data. Through comparison with the simultaneously recorded video data, we further verified the characteristic changes in the acceleration signal in the different behavioral states. The clear increase in three-axis acceleration when the cows performed other activities corresponded to their active physical activities. In the moving state, the acceleration value was irregular, which was consistent with the state of the cow moving at will. During rumination, the acceleration signal showed a relatively small fluctuation amplitude and tended to be stable, which was consistent with the more orderly and limited head movement during rumination. The different value ranges of these acceleration signals not only represented the distinction between different behavioral states, but their time series correspondence also provided accurate time markers for the automatic recognition of behavioral patterns.

#### 2.2.3. Classification Model Design

**Figure 7:**
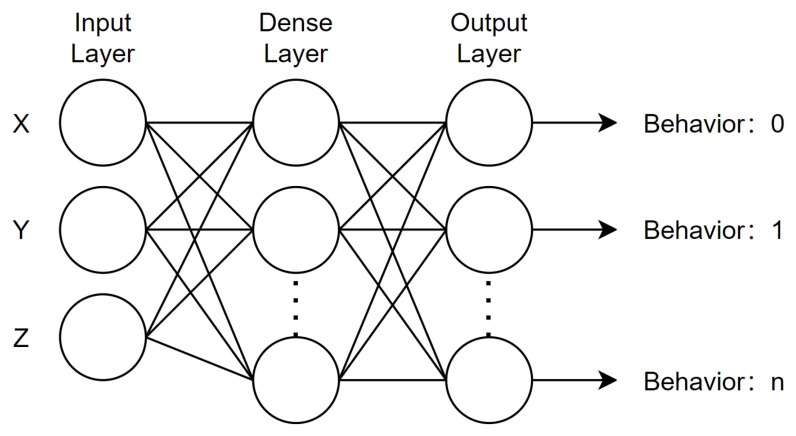
The LSTM model.

The long short-term memory network (LSTM) was selected as the cow behavior classification model [34]. The reason for choosing the LSTM model was to verify that the acceleration information collected by the equipment could still accurately analyze whether the cow behavior information corresponded to the data without relying on an overly complex model. This kind of classifier is widely used in behavior recognition research and has strong classification and recognition capabilities. The LSTM network can effectively process long-term series data through an internal gating mechanism and can capture long-term dependencies, which means that it can remember past information and use that information in subsequent time steps. Moreover, the LSTM model can extract rich contextual information from the input sequence and use that information in classification tasks to improve performance. The model structure is shown in Figure 7.

The first layer was an LSTM layer of 16 neurons, which were designed to process the input sequence. The second layer added a dense layer with 16 neurons and ReLU activation as a fully connected layer that introduced non-linearity, and it helped to learn complex patterns from the output of the LSTM. The last layer added another dense layer, which contained 3 neurons and a softmax activation function. This layer output the probability that the input belonged to one of three categories, thus making it suitable for multi-category classification tasks.

#### 2.2.4. Evaluation Index

This article used the recall rate and F1 Score coefficient to evaluate the behavior classification effect of the model. The formulas are as follows:

(1) $$\mathrm{Precision}=\frac{\mathrm{TP}}{\mathrm{TP}+\mathrm{FP}}$$

(2) $$\mathrm{Recall}=\frac{\mathrm{TP}}{\mathrm{TP}+\mathrm{FN}}$$

(3) $$F1=2\times \frac{\mathrm{precision}\times \mathrm{recall}}{\mathrm{precision}+\mathrm{recall}}$$

#### 2.2.5. Training Environment and Equipment Description

In this study, we used the Tensorflow framework. The training environment and equipment description are shown in Table 3.

## 3. Results

In summary, through in-depth analyses of the dairy cow behavior acceleration data, this study not only demonstrated clear differences in the acceleration characteristics between feeding, rumination, and other behaviors, but also revealed the biophysical mechanisms behind these differences. These findings provide a solid foundation for further developments of highly accurate cow behavior monitoring and automated management systems. We could clearly distinguish the subtle differences between the chewing and resting intervals of cows by analyzing the acceleration time series of the cow behavior, especially the changes in Z-axis acceleration. The specific fluctuation pattern of a Z-axis acceleration directly reflected the frequency and amplitude of the cow’s head moving up and down. This movement characteristic was particularly significant during rumination. In addition, through the observation of feeding behavior, we noticed synchronous fluctuations in the X-axis and Z-axis accelerations, which showed that the forward, backward, up, and down movements of the cow’s head were regular when eating, thus reflecting the periodicity of its eating behavior features. The number and times of the chewing when the cow ruminated could be determined by analyzing the peak value of the Z-axis acceleration.

The LSTM network model was used to accurately identify the three daily behaviors of cattle: eating, ruminating, and other behaviors. Moreover, the three metrics of precision, recall, and F1-score were found to be at high levels; in addition, all three values were similar. The model achieved a clear distinction between eating and rumination behaviors, and also reached the recognition level required in the industry. These results indicate that the LSTM network model can classify the various daily behaviors of cattle and provide technical support for smart breeding. When comparing the effects of the different time interception lengths (i.e., 3.2, 6.4, and 12.8 s) with respect to the accuracy of cow behavior recognition, 6.4 s was selected as the ideal time interception length, as the accuracy of the cow behavior recognition was at its highest. The accuracy of the cow behavior recognition at different interception times is shown in Table 4. The classification results and evaluation indicators of the LSTM model under 6.4 s are shown in Table 5.

**Figure 8:**
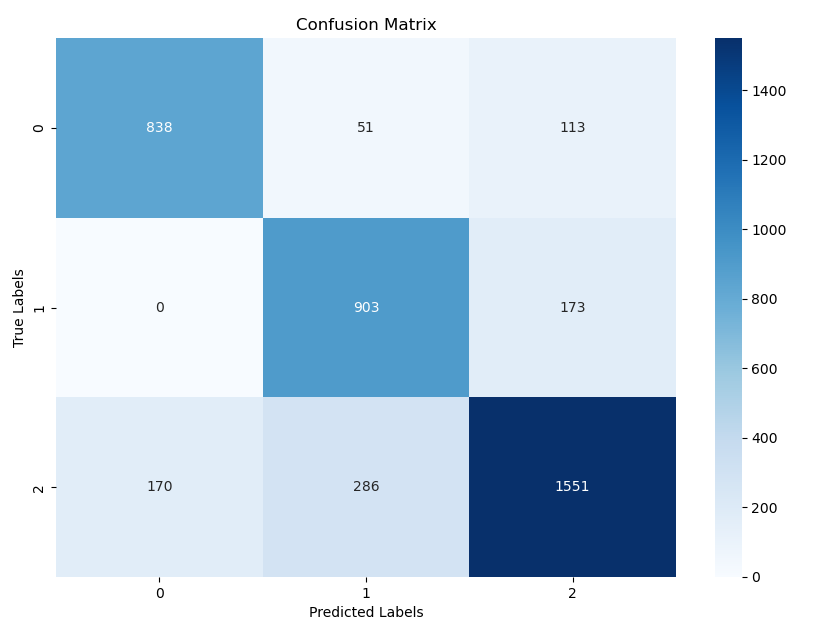
The LSTM confusion matrix.

The confusion matrix of the LSTM model is shown in Figure 8. Mutual misclassifications of the feeding and rumination behaviors was relatively rare, which indicated that the identification of these two behaviors was delivered with a high accuracy. However, these two types of behaviors were sometimes misclassified as other behaviors. The reason for this phenomenon may be that the cows were occasionally disturbed during the feeding and rumination process, such as when two cows were competing for food or when the surrounding environment changed. Cows suspend rumination or eating behavior and switch to other behaviors in these cases. Such behaviors are rare, but they will cause the behavior data to change from regular to irregular, resulting in the model misclassifying the behavior.

Personalized health and nutritional management can be achieved by continuously monitoring the physiological and behavioral indicators of a cow. For example, based on a cow’s activity level and feeding behavior, its dietary composition and supply can be adjusted to meet its specific nutritional needs. Behavioral changes in cows are often early signs of health problems. The rest duration, feeding, and rumination behavior can be determined in real-time through monitoring and analyzing cow activity. Our system can identify potential health issues, such as estrus, disease, or nutritional deficiencies early, thus allowing for timely intervention. The complex inner connections between physiological behavior and the health status of dairy cows can be revealed when a large quantity of collected data are obtained. These analysis results can provide farmers with scientific decision-making support through information on metrics such as optimal breeding time, health management measures, and nutritional adjustments. The discomfort and stress of the cows, in terms of wearing the equipment, should be reduced as much as possible, and they should be in line with the principles of animal welfare.

In comparison with traditional cow monitoring equipment, the first-generation pedometer used in the literature mainly follows the principle of a pedometer to record the number of steps and movements of the cow through the installation of sensors on a cow’s legs. The second-generation neck ring adopts more advanced technology and integrates 3D-accelerated sensing devices such as monitors and timers. In addition to accurate cow number identification, the second-generation neck ring can also collect various data such as activity level and estrus characteristics. However, these first two generations of dairy cow monitoring equipment alone cannot accurately identify the two key behaviors of dairy cows (i.e., eating and ruminating).

## 4. Discussion

Accurate monitoring of the physiological and behavioral indicators of dairy cows is essential to improve breeding efficiency and animal welfare. Although monitoring devices currently on the market, such as neck rings or pedometers, are effective in tracking animal activities, they are evidently deficient in comprehensively and accurately collecting key physiological indicators. In addition, such devices cannot provide detailed data on behaviors such as feeding and rumination. The nose ring’s close correlation with the cow’s mouth movements, as well as the AI model used, enabled it to capture and record behavior in greater detail in the pursuit of reliable data. The application of this equipment is expected to significantly improve the management efficiency and decision-making quality of the dairy farming industry, as well as promoting cost savings and efficiency improvements.

For our experiment, we collected acceleration data of the three daily behaviors of cattle (i.e., eating, ruminating, and other behaviors) via nose ring monitoring. Moreover, we then performed LSTM classification on the collected data. The results showed that the LSTM algorithm could identify the three behaviors of ruminating, eating, and other behaviors; having said that, however, there is currently no good way to distinguish between standing and lying-down behaviors. Thus, there is still a need to further improve the model’s recognition effect of these two behaviors, or a different, larger model should be used instead. The purpose of this experiment was to test the device on the cow’s nose and to establish whether there was a better recognition rate for cattle eating and rumination behavior; as such, the experiment was not performed with a larger model. At the same time, because cows do not stand and then lie down many times in a day, the data set had fewer categories for these two behaviors. Therefore, the model did not learn these two types of behaviors enough; thus, it could not accurately identify standing and lying behaviors. The results of this test can serve as a reference for improving the recognition level of cow behavior categories, as the degree of movement that a cow undertakes will change significantly when the cow in question has estrus or has developed hoof disease. Therefore, this algorithm can provide a reference and relevant theoretical support for the monitoring of cattle estrus and hoof disease.

There is still a long way to go in order to meet the demand for cow behavior detection systems. Although the work completed in this article met the proposed experimental requirements, there are still many problems that need to be addressed in the future. Some of the limitations of this study that are worthy of further exploration and research are as follows:

(1) Limited data set and sample size: This study involved a data set collected from seven cows, which, although sufficient for a preliminary analysis, may not fully represent the behavioral diversity of cows of different breeds, in different environments or health conditions. Expanding the data set will help to develop more powerful and general models.

(2) Single mode of behavior recognition: This research mainly relied on three-axis acceleration data for cow behavior recognition. While eating and rumination behaviors can be effectively captured, integrating other modalities such as acoustic signals, video analysis, or physiological sensors will increase the accuracy and range of behavioral identification, especially with respect to the complex behaviors that are difficult to distinguish through acceleration measurements alone.

(3) Focus on specific behaviors: Emphasizing eating and rumination behaviors is critical for health monitoring and productivity assessment. However, the inclusion of other behaviors such as social interactions, detection of heat, and signs of distress or disease can provide a more comprehensive understanding of dairy cow welfare and management needs.

(4) Energy efficiency and equipment design: although the equipment designed in this study is innovative, continued improvements in energy efficiency and wear resistance (e.g., reduced size, improved cow comfort, and so on) will further improve the application of this technology in actual agricultural settings, as well as in applicability and acceptance.

(5) Universality of the machine learning model: This study demonstrates the feasibility of using machine learning for behavioral classification based on acceleration data. Future work could explore the robustness of these models across different farms, species, and environmental conditions to ensure their broad applicability.

## 5. Conclusions

This study used a wearable cow nose ring device to collect cow movement data and also used deep learning algorithms to classify the different types of cow behaviors in order to help farm workers effectively determine the health level and estrus period of their cows. The three-axis acceleration data of the three identified behaviors of dairy cows—namely, eating, ruminating, and other behavior—were collected multiple times, and the long short-term memory network (LSTM) algorithm was used to successfully establish a behavior recognition model for dairy cows. The classification results showed that the LSTM model delivered accuracies of 81%, 86%, and 88% in terms of identifying the three behaviors of feeding, rumination, and other behaviors, respectively. Thus, the proposed model suggests a reference significance for wearable devices regarding cow behavior recognition.

For farmers, this equipment will be an effective tool for monitoring and preventing cow diseases. It can prevent the spread of cow diseases through identifying and curing them when they first appear. Such an approach could then reduce the economic losses caused by cow diseases in pastures and at the same time, it could improve the efficiency of detecting cow diseases, as well as helping to improve the welfare of dairy cows generally.

For large-scale farms, having too many cows makes it impossible for staff to immediately and accurately detect behavioral abnormalities and diseases in a certain cow. However, the proposed device can help to collect and analyze cow behavior data and can also aid in providing timely explanations of a cow’s behavior. Having more information on the behavioral conditions of cows can help farmers to manage their dairy herds.

Future research may involve collecting and analyzing behavioral data from a larger, more diverse population of dairy cows that are of different breeds, of different health status, and in different environmental conditions. This will help to develop more general and powerful behavior recognition models. Integrating other data modalities, such as visual (video analysis), auditory (sound analysis), or physiological (heart rate and body temperature) sensors, may help to significantly enhance the system’s capabilities, allowing for the identification of a wider range of behaviors and health conditions accurately. Expanding the range of recognized behaviors to include social interactions, estrus behavior, and the early signs of health problems could provide more comprehensive insights into farm management and animal welfare. Continued advances in sensor technology, energy harvesting, and materials science could lead to more efficient, durable, and cow-friendly wearable devices. This could include developing devices that are biodegradable or more environmentally friendly. Leveraging advances in artificial intelligence and machine learning, including deep learning and neural networks, could improve the accuracy and efficiency of behavioral classification. Exploring unsupervised or semi-supervised learning models may also reveal new insights into cow behavioral patterns. Developing systems that not only monitor and analyze cow behavior but also provide real-time alerts and recommendations for intervention could significantly improve farm management practices and animal welfare. Collaboration between technical experts, veterinarians, animal behaviorists, and farmers could help to develop more relevant, practical, and innovative solutions, which, in turn, will help to address the complexities of dairy cow behavior and welfare.

## Figures and Tables

**Table 1:** Cow behavior definitions.

Behaviors	Abbreviation	Description
Feeding behavior	FB	Defined as cows standing still with their legs extended and their necks bent to eat.
Rumination behavior	RB	Defined as a cow standing or lying still and chewing the cud repeatedly.
Other behaviors	OB	Defined as all behaviors other than eating and ruminating, including walking back and forth, tilting the head, standing still, sleeping, and so on.

**Table 2:** Types of behavior designated in numbers.

Behavior	Number
Feeding behavior	0
Rumination behavior	1
Other behaviors	2

**Table 3:** The training environment and equipment description.

Configuration	Parameter
Training framework	Python 3.10.9, Tensorflow Frame
Pre-trained model	LSTM Model
Operating system	Win 11
Development environment	IAR/pucharm
Computer configuration used in training and testing	Lenovo Rescuer 2023 model, R9-7945HX, RTX4060

**Table 4:** Accuracy of cow behavior recognition at different time interception lengths.

Time	Feeding	Rumination	Other
3.2	81	88	84
6.4	84	89	84
12.8	77	56	70

**Table 5:** Model classification results and evaluation indicators.

Behavior	Precision	Recall	F1-Score
Feeding	87	81	84
Rumination	87	91	89
Other	86	87	87

## Data Availability

Data were submitted to Kaggle and are available at https://www.kaggle.com/datasets/fandaoerji/cow-nose-ring-data-set (accessed on 1 November 2023).

## Abbreviations

The following abbreviations are used in this manuscript:

LSTM	Long short-term memory
CNRD	Cow nose ring data set

## Appendix A

**Table A1:** The SX1278 data packet loss rate.

Test Serial Number	Test Environment	Communication Distance (m)	Number of Data Packets Sent (Piece)	Number of Data Packets Received (Piece)	Packet Loss Rate (%)
1	Activity area	20	300	300	0.00
2	Activity area	40	300	299	0.03
3	Activity area	80	300	296	1.33
4	Activity area	100	300	292	2.66
5	Activity area	120	300	286	4.66
6	Activity area	150	300	283	5.66

## References

[1] Proudfoot K., Kull J., Krawczel P., Bewley J., O’Hara B., Donohue K., Pighetti G. (2021). Effects of acute lying and sleep deprivation on metabolic and inflammatory responses of lactating dairy cows. J. Dairy Sci..

[2] Barrell G.K. (2019). An appraisal of methods for measuring welfare of grazing ruminants. Front. Vet. Sci..

[3] Cramer M., Ollivett T., Stanton A. (2016). Associations of behavior-based measurements and clinical disease in preweaned, group-housed dairy calves. J. Dairy Sci..

[4] Goharshahi M., Azizzadeh M., Lidauer L., Steininger A., Kickinger F., Öhlschuster M., Auer W., Klein-Jöbstl D., Drillich M., Iwersen M. (2021). Monitoring selected behaviors of calves by use of an ear-attached accelerometer for detecting early indicators of diarrhea. J. Dairy Sci..

[5] Sepúlveda-Varas P., Weary D., Von Keyserlingk M. (2014). Lying behavior and postpartum health status in grazing dairy cows. J. Dairy Sci..

[6] Zheng Z., Zhang X., Qin L., Yue S., Zeng P. (2023). Cows’ legs tracking and lameness detection in dairy cattle using video analysis and Siamese neural networks. Comput. Electron. Agric..

[7] Hossain M.E., Kabir M.A., Zheng L., Swain D.L., McGrath S., Medway J. (2022). A systematic review of machine learning techniques for cattle identification: Datasets, methods and future directions. Artif. Intell. Agric..

[8] Hua Z., Wang Z., Xu X., Kong X., Song H. (2023). An effective PoseC3D model for typical action recognition of dairy cows based on skeleton features. Comput. Electron. Agric..

[9] Pavlovic D., Davison C., Hamilton A., Marko O., Atkinson R., Michie C., Crnojević V., Andonovic I., Bellekens X., Tachtatzis C. (2021). Classification of Cattle Behaviours Using Neck-Mounted Accelerometer-Equipped Collars and Convolutional Neural Networks. Sensors.

[10] Li C., Tokgoz K.K., Fukawa M., Bartels J., Ohashi T., Takeda K.i., Ito H. (2021). Data augmentation for inertial sensor data in CNNs for cattle behavior classification. IEEE Sens. Lett..

[11] Pereira G., Heins B., Endres M. (2018). Validation of an ear-tag accelerometer sensor to determine rumination, eating, and activity behaviors of grazing dairy cattle. J. Dairy Sci..

[12] Wolfger B., Timsit E., Pajor E., Cook N., Barkema H., Orsel K. (2015). Accuracy of an ear tag-attached accelerometer to monitor rumination and feeding behavior in feedlot cattle. J. Anim. Sci..

[13] Wu D., Han M., Song H., Song L., Duan Y. (2023). Monitoring the respiratory behavior of multiple cows based on computer vision and deep learning. J. Dairy Sci..

[14] Bresolin T., Ferreira R., Reyes F., Van Os J., Dórea J. (2023). Assessing optimal frequency for image acquisition in computer vision systems developed to monitor feeding behavior of group-housed Holstein heifers. J. Dairy Sci..

[15] Lodkaew T., Pasupa K., Loo C.K. (2023). CowXNet: An automated cow estrus detection system. Expert Syst. Appl..

[16] Dang N.H., Tran V.T., Dang T.H., Chung W.Y. (2023). Radio Frequency Energy Harvesting-Based Self-Powered Dairy Cow Behavior Classification System. IEEE Sens. J..

[17] Bokde N.D., Milkevych V., Nielsen R.K., Villumsen T.M., Sahana G. (2023). A novel approach for anomaly detection in dairy cow gas emission records. Comput. Electron. Agric..

[18] Shorten P., Hunter L. (2023). Acoustic sensors for automated detection of cow vocalization duration and type. Comput. Electron. Agric..

[19] Li G., Xiong Y., Du Q., Shi Z., Gates R.S. (2021). Classifying Ingestive Behavior of Dairy Cows via Automatic Sound Recognition. Sensors.

[20] Volkmann N., Kulig B., Hoppe S., Stracke J., Hensel O., Kemper N. (2021). On-farm detection of claw lesions in dairy cows based on acoustic analyses and machine learning. J. Dairy Sci..

[21] Abdanan Mehdizadeh S., Sari M., Orak H., Pereira D.F., Nääs I.d.A. (2023). Classifying Chewing and Rumination in Dairy Cows Using Sound Signals and Machine Learning. Animals.

[22] Bikker J., van Laar H., Rump P., Doorenbos J., van Meurs K., Griffioen G., Dijkstra J. (2014). Technical note: Evaluation of an ear-attached movement sensor to record cow feeding behavior and activity. J. Dairy Sci..

[23] Vu H., Chung H., Choi C., Kim Y. (2023). ETAG: An Energy-Neutral Ear Tag for Real-Time Body Temperature Monitoring of Dairy Cattle. Proceedings of the 29th Annual International Conference on Mobile Computing and Networking.

[24] Alsaaod M., Römer C., Kleinmanns J., Hendriksen K., Rose-Meierhöfer S., Plümer L., Büscher W. (2012). Electronic detection of lameness in dairy cows through measuring pedometric activity and lying behavior. Appl. Anim. Behav. Sci..

[25] de Mol R., André G., Bleumer E., van der Werf J., de Haas Y., van Reenen C. (2013). Applicability of day-to-day variation in behavior for the automated detection of lameness in dairy cows. J. Dairy Sci..

[26] Norring M., Häggman J., Simojoki H., Tamminen P., Winckler C., Pastell M. (2014). Short communication: Lameness impairs feeding behavior of dairy cows. J. Dairy Sci..

[27] Barker Z., Vázquez Diosdado J., Codling E., Bell N., Hodges H., Croft D., Amory J. (2018). Use of novel sensors combining local positioning and acceleration to measure feeding behavior differences associated with lameness in dairy cattle. J. Dairy Sci..

[28] Chen G., Li C., Guo Y., Shu H., Cao Z., Xu B. (2022). Recognition of Cattle’s Feeding Behaviors Using Noseband Pressure Sensor With Machine Learning. Front. Vet. Sci..

[29] Kröger I., Humer E., Neubauer V., Kraft N., Ertl P., Zebeli Q. (2016). Validation of a noseband sensor system for monitoring ruminating activity in cows under different feeding regimens. Livest. Sci..

[30] Zehner N., Umstätter C., Niederhauser J.J., Schick M. (2017). System specification and validation of a noseband pressure sensor for measurement of ruminating and eating behavior in stable-fed cows. Comput. Electron. Agric..

[31] González L., Bishop-Hurley G., Handcock R., Crossman C. (2015). Behavioral classification of data from collars containing motion sensors in grazing cattle. Comput. Electron. Agric..

[32] Nogoy K.M.C., Chon S.i., Park J.h., Sivamani S., Lee D.H., Choi S.H. (2022). High Precision Classification of Resting and Eating Behaviors of Cattle by Using a Collar-Fitted Triaxial Accelerometer Sensor. Sensors.

[33] Versluijs E., Niccolai L.J., Spedener M., Zimmermann B., Hessle A., Tofastrud M., Devineau O., Evans A.L. (2023). Classification of behaviors of free-ranging cattle using accelerometry signatures collected by virtual fence collars. Front. Anim. Sci..

[34] Sherratt F., Plummer A., Iravani P. (2021). Understanding LSTM network behaviour of IMU-based locomotion mode recognition for applications in prostheses and wearables. Sensors.

